# Antibody persistence and immune memory response following primary vaccination and boosting with live attenuated SA 14-14-2 Japanese encephalitis vaccine (CD-JEV) in Bangladesh: A phase 4 open-label clinical trial

**DOI:** 10.1016/j.jvacx.2022.100143

**Published:** 2022-02-05

**Authors:** K. Zaman, Md. Yunus, Asma B. Aziz, Jodi Feser, Jessica Mooney, Yuxiao Tang, Damon W. Ellison, Butsaya Thaisomboonsuk, Lei Zhang, Kathleen M. Neuzil, Anthony A. Marfin, G. William Letson

**Affiliations:** a International Centre for Diarrheal Disease Research, 68, Shaheed Tajuddin Ahmed Sarani, Mohakhali, Dhaka 1212, Bangladesh; b Center for Vaccine Innovation and Access, PATH, 2201 Westlake Ave, Suite 200, Seattle, Washington 98121, USA; c Armed Forces Research Institute of Medicine Sciences, 315/6 Rajvithi Rd., Bangkok 10400, Thailand; d Chengdu Institute of Biological Products Co., Ltd., 379, 3rd, Jinhua Road, Jinjiang District, Chengdu 61002, China

**Keywords:** Japanese encephalitis, Live attenuated SA-14-14-2, Vaccine, Persistence, Anamnestic response

## Abstract

**Introduction:**

Japanese encephalitis (JE) virus is one of the leading causes of viral encephalitis across temperate and tropical zones of Asia. The live attenuated SA 14-14-2 JE vaccine (CD-JEV) is one of three vaccines prequalified by the World Health Organization (WHO) to prevent JE. WHO currently recommends a single CD-JEV dose for infants in endemic settings. However, in the absence of long-term immunogenicity data, WHO has indicated a need for long-term immunogenicity studies to inform optimal dosing schedules and determine the need for booster doses.

**Methods:**

This Phase 4, open-label clinical study measured neutralizing antibody (NAb) titers in Bangladeshi children three and four years after primary CD-JEV vaccination and 7 and 28 days after a booster CD-JEV vaccination given four years after primary vaccination. The study also assessed the tolerability and safety of the booster dose. A NAb titer of ≥1:10 was considered seroprotective.

**Results:**

Of 560 children vaccinated between 10 and 12 months of age with CD-JEV three years earlier and enrolled in this study from 30 July 2015 through 03 January 2016, 52 (9.3%; 95% CI: 7.2–12.0) had a seroprotective titer at enrollment. One year later, of 533 children, 66 (12.4%; 95% CI: 9.9–15.5) had a seroprotective titer before receiving a booster dose. Of 524 children who received a booster CD-JEV dose, 479 (91.4%; 95% CI: 88.7–93.5) and 514 (98.1%; 95% CI: 96.5–99.0) were seroprotected 7 and 28 days later, respectively. The geometric mean titer (GMT) was 6 (95% CI: 6–6) at baseline, 105 (95% CI: 93–119) 7 days post-booster, and 167 (95% CI: 152–183) 28 days post-booster. No vaccine-associated neurologic adverse events or other serious adverse events were noted following the booster dose.

**Conclusions:**

Although most children did not have measurable antibody titers three and four years after a single primary CD-JEV dose, more than 90% of seronegative children had a strong anamnestic response within one week of a booster dose. This suggests that these children were immune despite the absence of measurable NAb prior to their booster.

ClinicalTrials.gov Identifier: NCT02514746.

## Highlights

• Despite a lack of measurable antibody four years after primary vaccination, the anamnestic response to a booster dose of live, attenuated SA 14-14-2 Japanese encephalitis vaccine indicates immunity persists.
• Live, attenuated SA 14-14-2 Japanese encephalitis vaccine is safe and well-tolerated.

## Introduction

Japanese encephalitis virus (JEV) is a major cause of neurologic morbidity and death in JE-endemic countries throughout south Asia, southeastern Asia, and the western Pacific [1]. In endemic areas of Vietnam, Sri Lanka, and Utter Pradesh (India) where JE vaccine has been introduced through national immunization programs, JEV still accounts for 26–62% of acute encephalitis syndrome (AES) cases where a viral etiology was identified [2], [3], [4]. Unfortunately, it is unclear from these studies whether these AES cases occurred in unvaccinated persons or vaccinated persons in whom immunity has waned.

Although this mosquito-borne flavivirus results in life-threatening encephalitis in roughly 1 out of every 250–1,000 JEV infections, 20–30% of persons with JEV encephalitis will die and up to 50% of survivors will have a significant neurologic impairment [5]. No antiviral treatment exists. As a result, seizure management, airway protection, ventilatory support, and other support measures are often required for treatment [6].

The global burden of disease has been estimated at roughly 68,000 cases per year, but that is likely an underestimate [5]. Vaccination with live and inactivated JE vaccines is a practical and cost-effective disease control measure [7], [8], [9], [10]. Because of the demonstrated effectiveness and impact of live attenuated SA 14-14-2 JE vaccine (CD-JEV), the World Health Organization (WHO) currently recommends its use as a single primary dose administered to children eight months of age or older [9]. However, WHO also recommends that additional research is needed to determine the long-term immunogenicity of CD-JEV, the potential need for a booster dose of CD-JEV, and the optimal dosing schedule if a booster dose is required [9].

In 2012, the International Centre for Diarrheal Disease Research, Bangladesh (icddr,b) and PATH conducted a double-blind, randomized controlled trial of a single dose of CD-JEV in Bangladeshi infants aged 10 to 12 months to determine the lot-to-lot consistency of CD-JEV manufactured in a newly constructed facility at the Chengdu Institute of Biological Products (CDIBP) and CD-JEV manufactured in the original facility [10]. In that study, seroprotection, defined as an anti-JEV neutralizing antibody (NAb) titer ≥ 1:10, ranged from 80.2 to 86.3% for all four vaccine lots, with an average geometric mean titer (GMT) of 56 measured 28 days after the primary vaccination [10]. Although long-term protection from CD-JEV is suggested by vaccine effectiveness (VE) case-control studies conducted five or more years after primary CD-JEV immunization, the long-term antibody kinetics following CD-JEV primary vaccination have not been fully studied [11], [12], [13], [14]. Similarly, the immune response to a CD-JEV booster dose has not been adequately studied [15]. This cohort of previously vaccinated Bangladeshi children provided an opportunity to understand better the anti-JEV NAb kinetics and antibody response following a booster CD-JEV dose. The current study aims were to describe the duration of immunity, to assess the anamnestic response, to inform CD-JEV dosing recommendations, and to allow greater generalizability of effectiveness studies that used the original facility product.

## Methods

### Study design and population

This was a Phase 4, open-label clinical trial involving a cohort of Bangladeshi children previously enrolled in a 2012 CD-JEV lot-to-lot consistency study (NCT 01635816) conducted by icddr,b and PATH, an international non-governmental organization based in Seattle, Washington, USA [10]. The study was conducted at two non-JE-endemic icddr,b study sites: Matlab, a rural area 50 km south of Dhaka, Bangladesh, and Mirpur, an urban site within Dhaka. The protocol and related documents were reviewed and approved by the icddr,b institutional review board and the Western Institutional Review Board on behalf of PATH. The study protocol is available online at clinicaltrials.gov NCT02514746.

Using local public health records and a demographic surveillance system, study staff recruited children vaccinated in the 2012 lot-to-lot consistency study who still lived in Matlab or Mirpur study areas. Criteria for inclusion in this study were: participation in the 2012 lot-to-lot consistency study; continued residence in study areas; and written informed consent from a parent or guardian allowing the child to give blood samples three and four years following the primary CD-JEV vaccination (before the booster vaccination), to receive a CD-JEV booster dose, and to provide blood samples 7 and 28 days following the booster dose. Children were excluded from this follow-up study if they had received another dose of JE vaccine within the four years following their primary CD-JEV vaccination; had been diagnosed with primary or acquired immunodeficiency including HIV infection; had received immunoglobulin, blood products, or immunomodulating or investigational medications within 90 days of the CD-JEV booster dose; or were severely malnourished, i.e., a weight-for-height z-score < -3 [16].

**Fig. 1:**
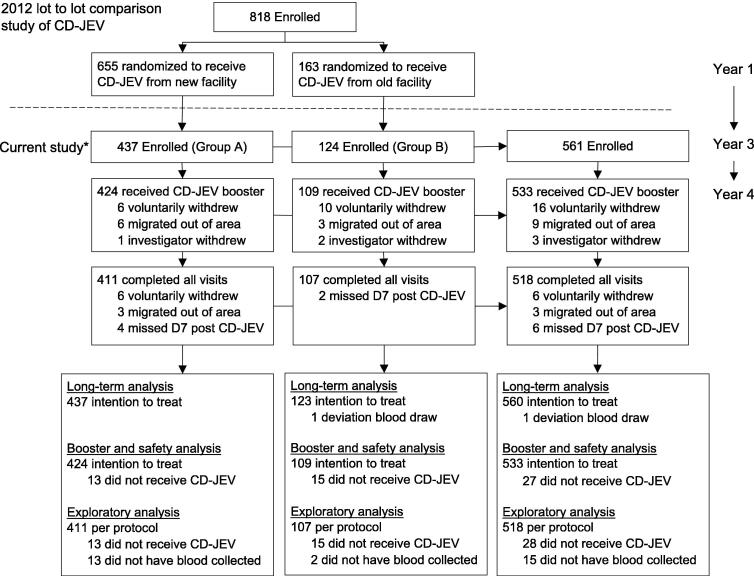
**Number of vaccine clinical study enrollees from original 2012 study and current long-term immunity and boosting study.** * Of the 818 enrolled in the 2012 lot to lot consistency study, 561 were still living in the area and interested in participating in the follow-up study (signed the informed consent document).

In the 2012 lot-to-lot study, 818 children between the ages of 10- to 12-months were randomized into four groups: three groups comprised of 655 children that received CD-JEV made in a newly constructed Good Manufacturing Practice (GMP) compliant facility and one group of 163 children that received CD-JEV produced at the original facility. Because there was no significant difference in the antibody response, the three groups that received vaccines from the GMP-compliant facility were combined in this study for the analyses. Thus, for the analyses in this study, the participants were divided into two groups based on whether they previously received vaccines from the newer GMP-compliant facility (Group A) or the original facility (Group B) (Fig. 1). No randomization or masking occurred in this study.

### Procedures

To determine the antibody status of study participants, 2 mL blood samples were collected three and four years after primary CD-JEV vaccination. After blood collection in Year 4, children received a subcutaneous injection in the upper right arm of 0.5 mL CD-JEV produced in the GMP-compliant facility (vaccine lot #201510C081-1 and diluent lot 201510C68, Chengdu Institute of Biological Products Co., Ltd, People's Republic of China). Blood samples were collected on Day 7 and Day 28 after the booster dose for serology testing. In addition, vaccinees were monitored for immediate reactions during the first 30 min, solicited injection site and systemic adverse reactions (AEs) for 7 days, and unsolicited AEs and serious adverse events (SAEs) for 28 days post booster dose. All safety events were identified or observed by study staff during home visits, clinic visits, and/or reported by a parent or legal guardian. Solicited and systemic AEs were assessed at home visits on Day 1 through Day 6 following vaccination and a fixed site clinic visit on Day 7 following vaccination. Information regarding unsolicited AEs occurring in the 28 days following vaccination were collected and recorded on a standard questionnaire during clinic visits. Events were graded for severity from mild to potentially life-threatening on predefined 1–4 scales based on functional assessment or magnitude of reaction and assessed for relatedness to vaccination by the investigators [17].

Serum samples were tested using plaque reduction neutralization test (PRNT) and JEV Immunoglobulin M (IgM) capture enzyme-linked immunosorbent assay (ELISA) at the Armed Forces Research Institute of Medicine Sciences, Bangkok, Thailand [18], [19]. PRNT data was expressed as the dilution causing 50% plaque reduction (PRNT-50) as extrapolated from a probit regression [20]. The assay was conducted in LLC-MK2 cells inoculated with JE SA-14-14-2 (0423-PDK-9) obtained from the Walter Reed Army Institute of Research. An anti-JE NAb titer ≥ 1:10 was considered both seropositive and seroprotective [21]. Using the following calculation, an anti-JE IgM ELISA assay result ≥ 40 ELISA units was considered positive [18].

$$100*(optical\;density\left[OD\right]\;sample_{av}-OD\;normal\;human\;serum{\left[NHS\right]}_{av}/\left(OD\;positive\;control_{av}-OD\;NHS_{av}\right))$$

### Outcomes

The primary study endpoints were seroprotection rates three and four years after primary vaccination and the anti-JE NAb GMT at these time points. The seroprotection rate was defined as the proportion of study participants with anti-JE NAb ≥ 1:10 as measured by PRNT-50 [21].

The secondary endpoints included seroprotection and seroconversion rates (defined as a change from seronegative to seropositive or a four-fold or greater rise in NAb titer for those seropositive at Year 4) 7 and 28 days after the CD-JEV booster vaccination, the anti-JE NAb GMT at these time points, and the GMT ratio between these time points and prior to the booster. In addition, safety endpoints included frequency counts and percentages of participants reporting immediate reactions within 30 min of vaccination, solicited and local adverse events within 7 days of vaccination, and unsolicited AEs or SAEs occurring within 28 days of booster vaccination.

As an exploratory objective to describe the kinetics of the booster response in the per-protocol population, study participants were categorized into two groups defined *a priori* (primed and unprimed) and adjusted to four groups *ad hoc* based on the results of anti-JE IgM ELISA and anti-JE PRNT. An anamnestic response was defined as a four-fold or greater rise in NAb titer within seven days of the booster for the exploratory objective [22]. The four groups were: 1) Primed—participants seronegative for anti-IgM prior to the booster and seven days after the booster with a four-fold or greater rise in NAb titer by Day 7 regardless of baseline NAb titer at Year 4; 2) Unprimed—participants seronegative for anti-IgM prior to the booster without a four-fold rise in NAb seven days after the booster but seroconverted by Day 28; 3) Seroprotected without anamnestic response—participants seropositive at baseline in Year 4 with less than a four-fold response at seven days after the booster; and 4) Non-responders—participants with no measurable NAb 28 days after the primary vaccination (from original 2012 study) and no measurable NAb at Year 4 after the primary vaccination, Day 7 after the booster vaccination, and Day 28 after the booster vaccination [10].

### Statistical analyses

Long-term antibody responses three and four years after primary CD-JEV vaccination and antibody responses following a booster dose of CD-JEV were analyzed descriptively using SAS v9.3. Participants who had at least one serology result in Year 3 or Year 4 were included in the intent to treat (ITT) analysis set for the long-term assessment (Fig. 1). The ITT set for the booster dose assessment included participants who received a CD-JEV booster dose and had at least one serology result on Day 7 or Day 28 after the booster vaccination. A per-protocol (PP) population for the booster dose analysis was a subset of the ITT population and defined as study participants who had serology results for Year 4, received a CD-JEV booster dose, and had serology results for both Day 7 and Day 28 after the booster vaccination.

The primary and secondary immunogenicity analyses were performed on the respective ITT populations and the exploratory analysis was performed on the PP booster dose assessment set. A descriptive safety analysis was conducted on the ITT population that received a CD-JEV booster.

The Wilson score method without continuity correction was used to obtain the 95% Confidence Interval (CI) for the proportion. The difference in proportions between Groups A and B was calculated along with the 95% CI using the Newcombe-Wilson method without continuity correction. GMTs and their 95% CI and the ratio of GMTs between Groups A and B with their 95% CI were calculated.

Booster response analyses were repeated on four subgroups defined by seroprotection status of participants at Year 4 post-primary CD-JEV vaccination (Table 4).

**Table 1:** Demographics and Participant Characteristics, ITT population.

	Group A (N = 437)	Group B (N = 124)	Total (N = 561)	P-value
Age (months), mean (SD)	47.7 (1.6)	47.8 (1.4)	47.7 (1.6)	0.3753

Gender at birth, n (%)				
Male	211 (48.3%)	59 (47.6%)	270 (48.1%)	0.9191
Female	226 (51.7%)	65 (52.4%)	291 (51.9%)	

Height (cm), mean (SD)	96.7 (4.1)	97.0 (4.0)	96.7 (4.0)	0.3459

Weight (kg), mean (SD)	13.7 (1.7)	13.7 (1.7)	13.7 (1.7)	0.9759

BMI for Age Z-score < -3 at Year 4, n (%)				
Yes	0 (0.0%)	0 (0.0%)	0 (0.0%)	1.0000
No	424 (97.0%)	109 (87.9%)	533 (95.0%)

**Table 2:** Seroprotection and GMT three and four years after primary vaccination with CD-JEV by originally assigned vaccine group, ITT population.

	n	% Seroprotection* (95% CI)	Difference (95% CI)	GMT (95% CI)	GMT Ratio (95% CI)
**Year 3**					
Group A†	437	9.2% (6.8–12.2)	−0.6% (-7.5–4.5)	6 (6–6)	1.0 (0.9–1.1)
Group B†	123	9.8% (5.7–16.3)		6 (5–6)	
Total	560	9.3% (7.2–12.0)		6 (6–6)	

**Year 4**					
Group A†	424	12.5% (9.7–16.0)	0.6% (-7.4–6.5)	6 (6–7)	1.0 (0.9–1.1)
Group B†	109	11.9% (7.1–19.3)		6 (5–7)	
Total	533	12.4% (9.9–15.5)		6 (6–6)	

* Seroprotection defined as anti-JEV neutralizing antibody titer ≥ 1:10.

† Group A = In 2012, received vaccine produced at the newer, GMP-compliant vaccine production facility; Group B = In 2012, received vaccine produced at an older vaccine production facility.

**Table 3:** Rise of anti-JE neutralizing antibody by 7 days and 28 days after CD-JEV booster vaccination given four years after primary CD-JEV vaccination by serostatus at Year 4, ITT population.

	Seronegative*	Seropositive	All
**Day 7 post-boost**			
n (%)	459 (87.6%)	65 (12.4%)	524 (100%)
% Seroprotection (95% CI)	90.4% (87.4–92.8)	98.5% (91.8–99.7)†	91.4% (88.7–93.5)
GMT (95% CI)	91 (80–104)	293 (229–375)	105 (93–119)
% ≥4-fold rise (95 %CI)	85.6% (82.1–88.5)	81.5% (70.4–89.1)	85.1% (81.8–87.9)
GMT Ratio‡ (95% CI)	18 (16–21)	12 (9–16)	17 (15–19)

**Day 28 post-boost**			
n (%)	461 (88.0%)	63 (12.0%)	524 (100%)
% Seroprotection (95% CI)	97.8% (96.1–98.8)	100% (94.3–100)	98.1% (96.5–99.0)
GMT (95% CI)	149 (135 – 165)	382 (319–457)	167 (152–183)
% ≥4-fold rise (95 %CI)	95.9% (93.7–97.3)	88.9% (78.8–94.5)	95.0% (92.8–96.6)
GMT Ratio‡ (95% CI)	30 (27–33)	16 (13–21)	28 (25–30)

* Seronegative defined as anti-JEV neutralizing antibody less than a 1:10 titer.

† Result due to variability of the assay. One participant had NAb titer of < 10 at Y3, 10 at Y4 prior to boost, <10 on D7 post booster, and 172 on D28.

‡ GMT post boost/GMT pre boost at Year 4.

**Table 4:** Geometric mean titers and change in antibody titer following CD-JEV booster dose by defined responder categories*, PP population.

	n (%)	Day 7 GMT (95% CI)	Day 7 GMT Ratio Post/Pre boost (95% CI)	Day 28 GMT (95% CI)	Day 28 GMT Ratio Post/Pre boost (95% CI)
Primed	440 (84.9%)	158 (144–174)	27 (25–29)	212 (196–230)	36 (33–39)
Unprimed	57 (11.0%)	8 (7–9)	2 (1–2)	45 (36–57)	9 (7–11)
Seroprotected without anamnestic response	12 (2.3%)	75 (35–163)	2 (2–3)	204 (138–301)	6 (3–9)
Non-responders	9 (1.7%)	5 (5–5)	1 (1–1)	5 (5–5)	1 (1–1)
Total	518 (100%)	105 (93–118)	17 (15–20)	168 (153–184)	28 (25–30)

* Primed: participants seronegative for anti-IgM prior to the booster and seven days after the booster with a four-fold or greater rise in NAb titer by Day 7; Unprimed: participants seronegative for anti-IgM prior to the booster without a four-fold rise in NAb seven days after the booster but seroconverted by Day 28; Seroprotected without anamnestic response: participants seropositive at baseline in Year 4 with less than a four-fold response at seven days after the booster; Non-responders: participants with no measurable NAb 28 days after the primary vaccination (from original 2012 study) and no measurable NAb at Year 4 after the primary vaccination, Day 7 after the booster vaccination, and Day 28 after the booster vaccination.

## Results

Of the 818 children enrolled in the 2012 study, 561 (68.6%) were enrolled in this study from 30 July 2015 through 03 January 2016. Of these 561 children, 560 (99.8%), 533 (95.0%), and 518 (92.3%) were included in the ITT long term, ITT booster dose, and PP booster dose analyses, respectively (Fig. 1). There were no significant differences in age, gender, or nutritional status between groups A (new facility) and B (original facility) (Table 1). To report on the full extent of the data, we are reporting on the ITT population where possible. However, 9 participants missed their Day 7 visit but returned for their Day 28 visit and another 9 missed their Day 28 visit but attended their Day 7 visit. This reduced the total to 524 rather than 533 in the immunogenicity analysis of the booster dose.

Three and four years after CD-JEV primary vaccination, 9.3% (95% CI: 7.2–12.0) and 12.4% (95% CI: 9.9–15.5) were seroprotected, respectively (Table 2). Four years after their primary CD-JEV vaccination, when comparing children who received vaccine manufactured in the newer CDIBP facility versus the older CDIBP facility, the difference between the proportion of children who were seroprotected was not significant (0.6% [95% CI: −7.4–6.5]).

In the total population, regardless of serostatus, 85.1% (95% CI: 81.8–87.9) had a four-fold or greater rise in NAb within seven days of the CD-JEV booster (Table 3). Overall, there was a 17-fold increase (95% CI: 15–19) in anti-JEV NAb by Day 7 and a 28-fold increase (95% CI: 25–30) in NAb by Day 28. Of 459 children who did not have measurable antibodies four years after their primary vaccination and prior to boosting, 393 (85.6% [95% CI: 82.1–88.5]) had a four-fold or greater increase in their NAb titers within seven days of their CD-JEV booster dose and were not demographically different from 53 out of 65 children (81.5% [95% CI: 70.4–89.1]) who had measurable NAb before their booster dose (Table 3).

Of 518 children for whom Year 4 (post-primary), Day 7 (post-booster), and Day 28 (post-booster) serology results were available, 440 (84.9%) were primed responders with a 27-fold increase in anti-JEV NAb (95% CI: 25–29) by Day 7 after their booster and a 36-fold increase (95% CI: 33–39) by Day 28 (Table 4). Additionally, 57 (11.0%) were unprimed responders with GMT estimated to be 8 (95% CI: 7–9) and 45 (95% CI: 36–57) on Day 7 and 28 after their CD-JEV booster dose, respectively. Although this slower rise of NAb titers is more consistent with a primary immune response to CD-JEV vaccination, no anti-JEV IgM antibody was identified in any of these 57 children. Interestingly, nine children (1.7%) did not show any rise in anti-JEV NAb by Day 28 post-booster. Upon review of the results from the 2012 study of primary vaccination, none of these nine children made NAb by Day 28 post-primary vaccination either.

The safety analysis set included all enrolled participants who received a booster dose (N = 533). Briefly, 4 (0.8%) participants experienced an immediate reaction (all mild), 2 (0.4%) experienced a local reaction within 7 days (all mild), 19 (3.6%) reported a systemic reaction within 7 days, 83 (15.6%) reported an unsolicited adverse event within 28 days of which 4 (0.8%) were solicited events (pain [2], fever [1], and irritability [1]) considered related to vaccination that were ongoing after 7 days (Table 5). All related unsolicited events resolved within 2–3 days after onset. Of the systemic reactions, the most common was fever (17/23, 74%) of grade 1 (axillary temperature 37.5–37.9 °C, n = 13) or grade 2 (axillary temperature 38.0–38.4 °C, n = 4). No SAEs or unsolicited reports of encephalitis, meningitis, or other severe neurologic illnesses were reported in the 28 days post-booster.

**Table 5:** Summary of safety measures by maximum severity*, safety population (N = 533).

	None n (%)	Grade 1 n (%)	Grade 2 n (%)	Grade 3 n (%)	Grade 4 n (%)
**Immediate reaction**	529 (99.2%)	4 (0.8%)	**–**	**–**	**–**
Injection site pain	532 (99.8%)	1 (0.2%)	**–**	**–**	**–**
Injection site swelling	529 (99.2%)	4 (0.8%)	**–**	**–**	**–**
**Solicited systemic reactions**	514 (96.4%)	15 (2.8%)	4 (0.8%)		
Change in eating habits	531 (99.6%)	2 (0.4%)	**–**	**–**	**–**
Irritability	532 (99.8%)	1 (0.2%)	**–**	**–**	**–**
Vomiting	532 (99.8%)	1 (0.2%)	**–**	**–**	**–**
Fever	518 (97.2%)	11 (2.1%)	4 (0.8%)	**–**	**–**
**Unsolicited adverse events**†	450 (84.4%)	70 (13.1%)	12 (2.3%)	1 (0.2%)	**–**
Ear and labyrinth disorders	531 (99.6%)	2 (0.4%)	**–**	**–**	**–**
Gastrointestinal disorders	525 (98.5%)	7 (1.3%)	1 (0.2%)	**–**	**–**
General disorders and administration site issues	520 (97.6%)	12 (2.3%)	1 (0.2%)	**–**	**–**
Infections and infestations	494 (92.7%)	31 (5.8%)	7 (1.3%)	1 (0.2%)	
Injury, poisoning and procedural complications	529 (99.2%)	2 (0.4%)	2 (0.4%)	**–**	**–**
Investigations	532 (99.8%)	1 (0.2%)	**–**	**–**	**–**
Metabolism and nutrition disorders	532 (99.8%)	1 (0.2%)	**–**	**–**	**–**
Nervous system disorders	532 (99.8%)	1 (0.2%)	**–**	**–**	**–**
Psychiatric disorders	532 (99.8%)	1 (0.2%)	**–**	**–**	**–**
Respiratory, thoracic and mediastinal disorders	515 (96.6%)	17 (3.2%)	1 (0.2%)	**–**	**–**
Skin and subcutaneous tissue disorders	529 (99.2%)	2 (0.4%)	**–**	**–**	**–**

* Grade 1 = Mild; Grade 2 = Moderate; Grade 3 = Severe; Grade 4 = Potentially Life-Threatening.

† Per system organ class as defined by the Medical Dictionary for Regulatory Activities (MedDRA) v19.0.

## Discussion

In this population of Bangladeshi children who received a single dose of CD-JEV in their first year of life, nearly 88% did not have detectable seroprotective anti-JEV neutralizing antibody titers four years later. When considering the need for a CD-JEV booster dose, it is crucial to determine whether children without measurable NAb titers several years after primary vaccination are susceptible or whether they can rapidly mount a sufficient immune response to stop viral proliferation when infected with a wild-type JEV. In this study, when those seronegative children were given a booster dose of CD-JEV, the majority (86%) had a four-fold or greater NAb response within one week of their booster dose similar to the 82% in seropositive children. In addition, the booster CD-JEV dose was safe and well-tolerated with an overall safety profile similar to that reported when CD-JEV was given as a primary dose to these same children four years earlier [10]. These results are similar to the immune response shown with the live attenuated recombinant (chimeric) JE vaccine (IMOJEV, Sanofi Pasteur), wherein seroprotection had fallen substantially by year five following primary immunization but a robust anamnestic response with boosting at five-year follow-up was seen [23]. Because the incubation period of JEV is 5–15 days, despite this vigorous response, the question remains whether this response is rapid enough to protect against wild-type JEV infection or whether NAb needs to be present before JEV infection to achieve protection [24].

In contrast, roughly 11% of the children had no measurable antibody prior to boosting and had a slower and lower NAb rise in the first week after boosting. This antibody response appears to be the recapitulation of the primary immune response as indicated by little or no NAb titers at Day 7 but protective antibody titers by Day 28. In this study, the Day 28 GMT for this group of children was 45 (95% CI: 36–57), comparable to NAb titers resulting from primary vaccination in the original 2012 study [10]. None of these children developed anti-JEV IgM antibodies within seven days of the booster vaccination, raising concerns about their ability to control a JEV infection upon exposure effectively. Although the development of IgM antibody may accompany the primary immune response, the lack of IgM here may be due to not measuring IgM antibody at the optimal time, IgM titer that was below the threshold for detection, or highly variable IgM responses as seen in earlier studies of the primary immune response to CD-JEV and with 17D yellow fever (YF) vaccine [25], [26], [27]. For example, in one study of a 17D YF vaccine booster given two years after primary vaccination, the nonprimed NAb and IgG antibody responses were not associated with an IgM response [26]. In another study, persons revaccinated more than ten years after their primary 17D YF vaccination developed anti-YF IgM antibody [27].

The slow rise in NAb following a CD-JEV booster in 11% of seronegative children suggests that a proportion of CD-JEV-vaccinated children living in areas of low endemicity may not be able to mount a rapid, protective immune response to protect them against wild-type JEV infection. In addition, it is unknown whether the proportion of seronegative, unprimed children will continue to increase over time following primary vaccination without a booster. Consequently, the full benefits of a CD-JEV booster dose in this population remain unknown.

Currently, WHO does not recommend a booster dose among CD-JEV recipients. The two largest users of CD-JEV, China and India, administer multiple CD-JEV doses in their national immunization programs, but this is generally intended to overcome low JE vaccination coverage as opposed to specifically enhancing the effectiveness or performance of the vaccine [15]. Studies of the potential immunologic benefit of a booster CD-JEV dose are limited. In a 1993 study of vaccine effectiveness (VE) of CD-JEV in rural Sichuan province, the VE of a single CD-JEV dose was 80% (95% CI: 44–93%), whereas the VE for children given two CD-JEV doses one year apart was 98% (95% CI: 86–100%) [14]. In a 2006 study in an area of low JE endemicity in Nepal, 44 (64%) of 69 children given a primary CD-JEV vaccination in 2000 were still seroprotected in 2005. Of 17 seronegative children in this Nepali population given a booster CD-JEV dose in 2006, 13 (77%) developed a strong anamnestic antibody response within seven days of the booster (GMT 169; titer range: 38–2173), showing that these children were primed and likely still immune to JEV infection [15]. Of the remaining four children in this small study, three did not develop any anti-JEV antibody within 28 days of boosting and one did not have measurable antibody seven days after the booster but did develop a moderately high antibody titer 21 days later. The populations in our study and these two studies lived in areas of low JE endemicity, which could increase the potential benefit of a booster dose of CD-JEV. Whether these immunologic benefits would also be realized in areas of high JE endemicity is unknown.

Nearly 2% of children in this study did not have measurable NAb at Year 4 and did not develop an immune response within four weeks of a booster dose. In the previous 2012 study, these same children did not develop antibodies within four weeks of their primary CD-JEV vaccination [10]. Given that these children failed to show a primary immune response after two different CD-JEV vaccinations given four years apart while most children were seroprotected or had an anamnestic antibody response, it is unlikely this failure to immunize is due to factors related to vaccine administration or the age at vaccination. Instead, it is likely due to host factors, such as host genetics, immune status, health, nutritional status, or a robust innate immune response that does not allow viral proliferation following vaccination, thus blocking immunization. Non-responders have been described in other flavivirus vaccine recipients. From 1973 to 2008, Brazil reported seven 17DD yellow fever vaccine failures, of which five were classified as primary vaccine failures (i.e., development of yellow fever in persons vaccinated <10 years earlier) [28]. It is unknown whether these vaccine failures represent people who never developed a protective immune response, had a delayed initial response that rapidly waned, or had adequate antibody titers but inadequate innate or adaptive cellular immune responses.

The 2015 WHO position paper on Japanese encephalitis vaccines recommends a single primary dose administered to children at least eight months of age and that the need for a booster CD-JEV dose in endemic settings still needs to be determined [9]. Neither of the two Bangladesh sites in this study is considered JE-endemic. Whether the seroprotection rates at four years after primary vaccination would have been higher in an endemic area due to continuous antigenic stimulation is presumed but unknown. This study shows that most children vaccinated with CD-JEV within the preceding four years and living in non-endemic areas will mount a strong and rapid immune response to JEV infection, regardless of measurable anti-JEV NAb titers prior to infection and without continuous antigenic stimulation.

Determining whether a booster dose of CD-JEV is immunologically needed will be difficult. Despite the high proportion of seronegative children several years after CD-JEV primary vaccination in this study, the strong anamnestic response to a booster dose is consistent with the high-level of vaccine effectiveness and high vaccine impact observed in earlier studies of CD-JEV. Even assuming that a large proportion of vaccinated children become seronegative over time, the very rapid and profound immune response in primed children is consistent with the 96% VE and 78% disease reduction five years after vaccine introduction seen in Nepal [11], [12] or the 79% VE six years after vaccine introduction seen in Gorakpur, India [13]. Primary CD-JEV vaccination resulted in long-term immune memory for most children in this study, as demonstrated by the marked anamnestic response. Assuming that the kinetics of viral proliferation following vaccination with the SA 14-14-2 JEV strain are similar to that of a wild-type JEV infection, the immune response of most seronegative children following a single primary vaccination dose of CD-JEV should provide adequate protection against JEV, even in non-endemic areas, for the majority of primary vaccinees. However, the findings in this study should be considered together with the current wide acceptance of anti-JEV NAb antibody titer ≥ 1:10 as an immune correlate of protection. This threshold titer was not present in 9% of our study subjects who were not “seroprotected” within seven days of boosting. The findings in this study are also consistent with lower VE estimates that may reflect a sub-population that was not adequately primed for an anamnestic response following a single dose of CD-JEV. As seen in the 1993 VE study from Sichuan Province, the effectiveness can be greatly improved with a booster at one year after primary immunization [14].

This study had limitations. For administrative and logistic reasons, this study did not include a control group of previously unvaccinated four- and five-year-old children for comparison. In addition, this study used a different laboratory than the one used in the 2012 study of these same children. Comparing NAb titers across studies when the assays are performed in different laboratories has many challenges due to differences in the lab methods and the inherent variability of PRNT [29]. While acknowledging these limitations, the GMT in this study is greater than the GMT reported for these same children measured 28 days after their primary CD-JEV vaccination, which was 56 (95% CI: 50–64) [10].

In summary, despite low rates of seroprotection prior to a booster dose with CD-JEV, 91% of children were seroprotected within 7 days of boosting and 98% were seroprotected within 28 days. We believe the high level of seroprotective response and immunologically defined anamnestic response in 85% of children show long-standing immune memory in children who have received a single primary dose of CD-JEV. These findings support the current WHO recommendation of a single dose of this vaccine without boosting. Still, about 11% of children had a less vigorous antibody response, and there may be concern that their response to wild-type JEV infection may not be adequate to provide protection. In addition, this is the first study to show that nearly 2% of children receiving two doses of CD-JEV did not mount a primary immune response on either occasion.

While informative, the immunogenicity data from this study are not sufficient to shape a booster dose strategy for CD-JEV or to revise WHO booster policy regarding CD-JEV. If up to 11% of vaccinated children are not immune four years after their primary CD-JEV dose, these data would need to be integrated with modeling, health economics, and similar antibody persistence/boosting studies performed among children living in endemic areas to shape such policies. In addition, long-term vaccine effectiveness studies and robust investigations into vaccine failures are critical to inform this policy and strongly recommended. More importantly, similar to what was done to determine the need for measles vaccine boosters in the United States in the late 1980s, epidemiologic investigations of JE and AES of unknown etiology in children previously vaccinated with CD-JEV should be performed to identify breakthrough cases of JE and the magnitude of the problem [30].

## CRediT authorship contribution statement

**K. Zaman:** Conceptualization, Methodology, Validation, Investigation, Resources, Data curation, Writing – review & editing, Supervision, Project administration. **Md. Yunus:** Conceptualization, Methodology, Validation, Investigation, Resources, Data curation, Writing – review & editing, Supervision, Project administration. **Asma B. Aziz:** Conceptualization, Methodology, Validation, Investigation, Resources, Data curation, Writing – review & editing, Supervision, Project administration. **Jodi Feser:** Conceptualization, Methodology, Validation, Formal analysis, Resources, Data curation, Writing – original draft, Writing – review & editing, Visualization, Supervision, Project administration. **Jessica Mooney:** Methodology, Validation, Resources, Data curation, Writing – review & editing, Visualization, Supervision, Project administration. **Yuxiao Tang:** Conceptualization, Methodology, Validation, Formal analysis, Resources, Data curation, Writing – original draft, Writing – review & editing, Visualization, Supervision, Project administration. **Damon W. Ellison:** Conceptualization, Methodology, Validation, Formal analysis, Investigation, Resources, Data curation, Writing – review & editing, Supervision, Project administration. **Butsaya Thaisomboonsuk:** Conceptualization, Methodology, Validation, Formal analysis, Investigation, Resources, Data curation, Writing – review & editing, Supervision, Project administration. **Lei Zhang:** Conceptualization, Writing – review & editing. **Kathleen M. Neuzil:** Conceptualization, Methodology, Writing – review & editing, Visualization, Funding acquisition. **Anthony A. Marfin:** Conceptualization, Methodology, Validation, Resources, Data curation, Writing – original draft, Writing – review & editing, Visualization, Supervision, Project administration, Funding acquisition. **G. William Letson:** Conceptualization, Methodology, Validation, Resources, Data curation, Writing – original draft, Writing – review & editing, Visualization, Supervision, Project administration.

## Funding

This work is based on research funded in part by the 10.13039/100000865Bill & Melinda Gates Foundation. The findings and conclusions contained within are those of the authors and do not necessarily reflect positions or policies of the Foundation.

## Disclaimer

This material has been reviewed by the Walter Reed Army Institute of Research. There is no objection to its presentation and/or publication. The opinions or assertions contained herein are the private views of the author, and are not to be construed as official, or as reflecting true views of the Department of the Army or the Department of Defense. The investigators have adhered to the policies for protection of human subjects as prescribed in AR 70–25.

## Registrations

ClinicalTrials.gov Identifier: NCT02514746.

## Declaration of Competing Interest

The authors declare the following financial interests/personal relationships which may be considered as potential competing interests: L Zhang is employed by the manufacturer of the vaccine, Chengdu Institute of Biological Products Co., Ltd. All other authors declare no competing interests.
